# Integrated structure-based protein interface prediction

**DOI:** 10.1186/s12859-022-04852-2

**Published:** 2022-07-25

**Authors:** M. Walder, E. Edelstein, M. Carroll, S. Lazarev, J. E. Fajardo, A. Fiser, R. Viswanathan

**Affiliations:** 1 Department of Chemistry, Yeshiva College, Yeshiva University, New York, NY 10033 USA; 2 Department of Systems and Computational Biology, Albert Einstein College of Medicine, Bronx, NY 10461 USA

**Keywords:** Protein–protein interaction, Interface prediction, Structure-based method

## Abstract

**Background:**

Identifying protein interfaces can inform how proteins interact with their binding partners, uncover the regulatory mechanisms that control biological functions and guide the development of novel therapeutic agents. A variety of computational approaches have been developed for predicting a protein’s interfacial residues from its known sequence and structure. Methods using the known three-dimensional structures of proteins can be template-based or template-free. Template-based methods have limited success in predicting interfaces when homologues with known complex structures are not available to use as templates. The prediction performance of template-free methods that only rely only upon proteins’ intrinsic properties is limited by the amount of biologically relevant features that can be included in an interface prediction model.

**Results:**

We describe the development of an integrated method for protein interface prediction (ISPIP) to explore the hypothesis that the efficacy of a computational prediction method of protein binding sites can be enhanced by using a combination of methods that rely on orthogonal structure-based properties of a query protein, combining and balancing both template-free and template-based features. ISPIP is a method that integrates these approaches through simple linear or logistic regression models and more complex decision tree models. On a diverse test set of 156 query proteins, ISPIP outperforms each of its individual classifiers in identifying protein binding interfaces.

**Conclusions:**

The integrated method captures the best performance of individual classifiers and delivers an improved interface prediction. The method is robust and performs well even when one of the individual classifiers performs poorly on a particular query protein. This work demonstrates that integrating orthogonal methods that depend on different structural properties of proteins performs better at interface prediction than any individual classifier alone.

**Supplementary Information:**

The online version contains supplementary material available at 10.1186/s12859-022-04852-2.

## Background

Proteins execute their diverse range of biological functions through interactions with other proteins and small molecules, which lead to the formation of larger scale protein interaction networks (interactomes). A protein’s function in the interactome is characterized by its interaction partners. Knowledge of a protein’s binding interfacial residues is essential for elucidating the molecular mechanism by which it performs its function, for determining the functional effect of mutations, as well as for designing drugs to disrupt a biological network by targeting a specific protein–protein interaction (PPI) [1].

Experimental techniques commonly employed to determine the structure of protein complexes at atomic-scale resolution include X-ray crystallography [2, 3] nuclear magnetic resonance (NMR) spectroscopy [4], and cryo-electron microscopy (cryo-EM) [5]. Information about interface residues can also be obtained by alanine scanning mutagenesis experiments [6, 7] or various footprinting experiments, such as hydrogen/deuterium exchange or hydroxy radical footprinting [8]. Since X-ray crystallography requires crystallization of specimens, it can only be used to analyze non-dynamic complexes and often under non-physiological conditions. While NMR does not require samples to be crystallized it is limited to determining the structure of smaller proteins with molecular weight around 20 kDa. Cryo-EM allows the structure of proteins to be visualized while they are in an aqueous environment, which resembles their native intracellular environment. However, cryo-EM experiments also require cryogenic temperatures, usually lower than −135 °C, to maintain the sample in a vitrified state. More importantly, all these approaches require a prior knowledge of a cognate binding partner. Due to the limitations, low-throughput, and costly nature of experimental approaches, computational prediction methods are employed to streamline the process of identifying the interfacial residues of proteins.

Prediction methods can rely solely upon query proteins’ sequence information (sequence-based), or they can also be based on query proteins’ 3-dimensional structure (structure-based). Sequence-based methods can be implemented on almost any protein, whereas structure-based approaches are limited to proteins with known structures in the Protein Data Bank [9]. Sequence-based methods are based on finding relationships between the likelihood of a residue to be interfacial and its sequence-related properties like hydrophobicity distribution, interface propensity, and physico-chemical properties [10, 11]. In a typical sequence-based method, overlapping sequence segments of the query protein are obtained by using a sliding window of width ranging from 3 to 30 residues [12] with target residue at the center of these segments. Each segment is assigned a feature vector based on properties of amino acids. These feature vectors from a set of proteins with known interface residues are used to train machine learning algorithms like random forest [13] or support vector machine [13–19]. The trained models are then used in a binary classification problem to predict the interfacial residues of each query protein using its feature vectors as inputs.

Structure-based approaches depend upon the availability and quality of 3D structures, and most of these methods outperform sequence-based methods [20]. There are two main classes of structure-based methods, which are referred to here as “template-free” or “template-based” approaches. Template-free methods train machine learning algorithms on a dataset of experimentally determined protein complex structures to create a model that relates sequence and structural features with the likelihood for residues to be at the binding interface. These template-free methods may include sequence features such as hydrophobicity, propensity of amino acids to be at an interface, physico-chemical properties, evolutionary conservation, and structural features such as secondary structure, solvent-accessible surface area, and geometric shape [10, 14, 21, 22]. While template-free methods have been steadily enhanced over the past 20 years, their future improvement appears to be limited because further combination of existing features and classifiers has little impact on performance [10, 23]. In contrast, template-based approaches predict interfacial residues by mapping interface information onto the query protein from its homologues or structural neighbors with known complex structures [1]. The drawback of template-based methods is that their effectiveness is dependent upon the existence of homologues or structural neighbors that have had their complex structure experimentally determined [10].

Methods that require the structure of both proteins in a complex to make a prediction are called partner-specific, and methods that can make interface predictions on individual unbound proteins are referred to as partner-independent. Some template-free methods, like ISPRED4 [24], are partner-independent, while other template-free approaches, like Daberdaku et al. [25] 3D Zernike descriptor method, are partner-specific. Currently there are several template-based methods that depend on known structural neighbors for predicting interfaces. Some of these methods, like PS-HomPPI [26], are partner-specific. Other template-based methods, like PredUs 2.0 [1] and PriSE [27], do not need information about the binding partner. In order to be more generic, we focused on methods that can make predictions of interface residues without the knowledge of the cognate partner protein’s structure.

A few meta-methods that integrate different interface predictors to generate a consensus prediction have also been developed. Meta-PPISP is one such meta-method that combines the predictors cons-PPISP [28], Promate [29], and PINUP [30] through linear regression analysis [31]. The success of a meta-method is contingent on the input predictors contributing orthogonal information to the consensus model [10]. The inputs for meta-PPISP have limited orthogonality because it combines three template-free approaches, and it does not consider inputs from template-based or docking-based approaches. Additionally, meta-PPISP employed linear regression analysis for method combination, which is likely less robust than using more complex tree-based regression models.

Both classes of structure-based methods described above, template-free and template-based, have strengths and limitations. To take advantage of the successes of both these types of methods, we aimed to create a meta-method that integrates the orthogonal template-based, template-free, and docking-based predictors. Among the available template-based methods that are not partner-specific, we chose PredUs 2.0, as the webserver was readily available and could be automated on a large dataset. For a similar reason, we chose ISPRED4 [24] as the template-free method. We have recently shown that protein interfaces can be predicted effectively using a docking-based approach without knowledge of the binding partner [32], and we refer to this method as DockPred. Our goal is to improve the PPI binding interface predictions made by DockPred by integrating this method with two other orthologous approaches, PredUs 2.0 (template-based) [1] and ISPRED4 (template-free) [24].

In this work, we present an **I**ntegrated **S**tructure-based **P**rotein **I**nterface **P**rediction (ISPIP) method that generates an enhanced consensus prediction by integrating the predictive strengths of orthogonal template-based (PredUs 2.0), template-free (ISPRED4), and docking-based (DockPred) predictors. To develop ISPIP, regression models of varying complexity were trained on the three input classifiers’ interface scores for a training set of query proteins with known complex structures. Not only is ISPIP’s consensus predictor significantly enhanced relative to DockPred and the other input predictors, it also outperforms a previous consensus predictor (meta-PPISP) and a complex structure-based method (VORFFIP).

## Results

### Enhanced interface prediction of ISPIP model

**Fig. 1:**
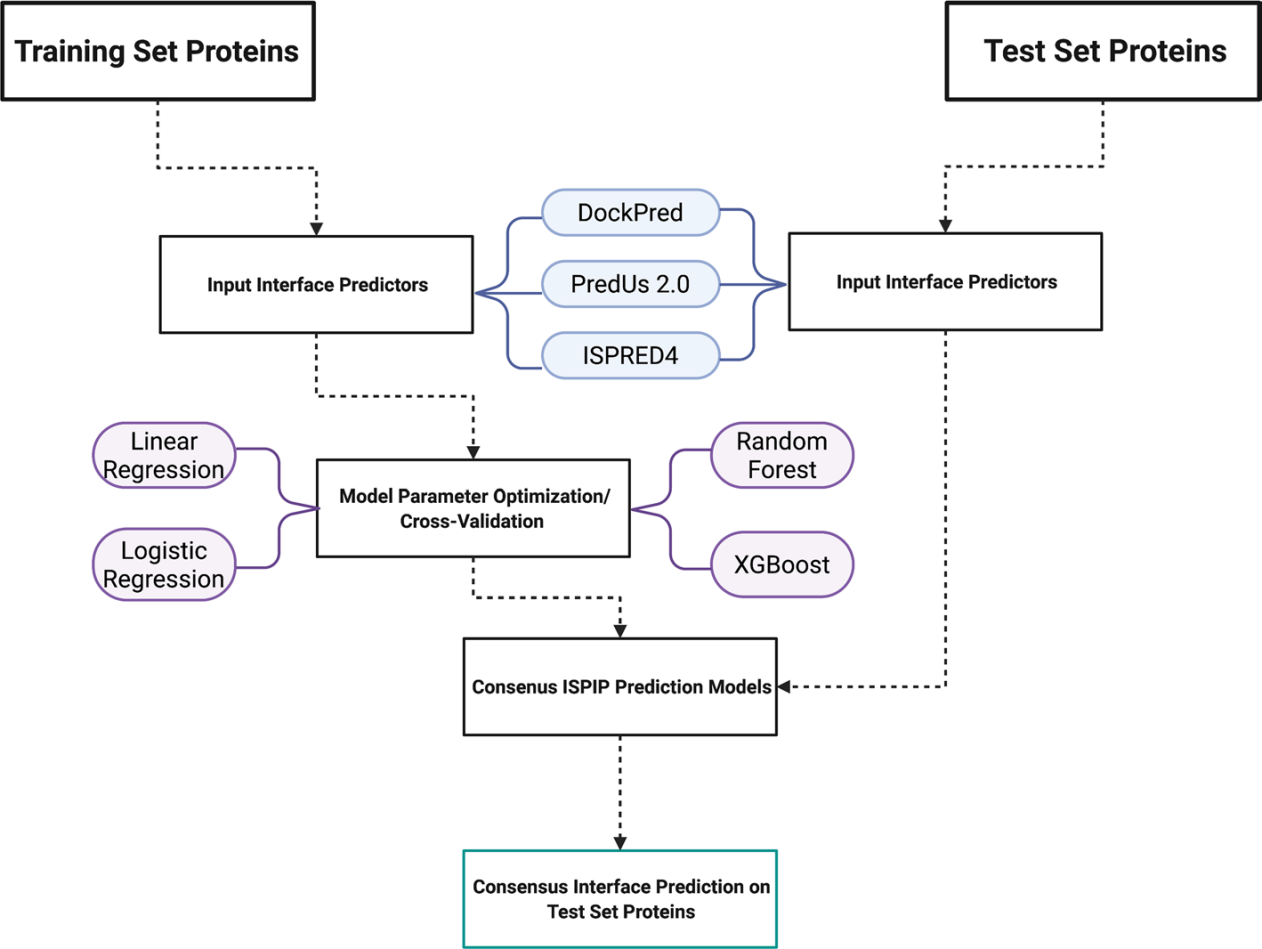
Flowchart of ISPIP Methodology: ISPIP’s classification models are generated through training on the interface likelihoods of the three input predictors. (Created with BioRender.com)

When designing ISPIP, we aimed to develop a model that could predict query proteins’ interfacial residues more effectively than the three orthogonal input predictors (DockPred, ISPRED4, PredUs2.0) alone. Figure 1 shows a flow chart of the methodology used to combine the three individual classifiers.

**Fig. 2:**
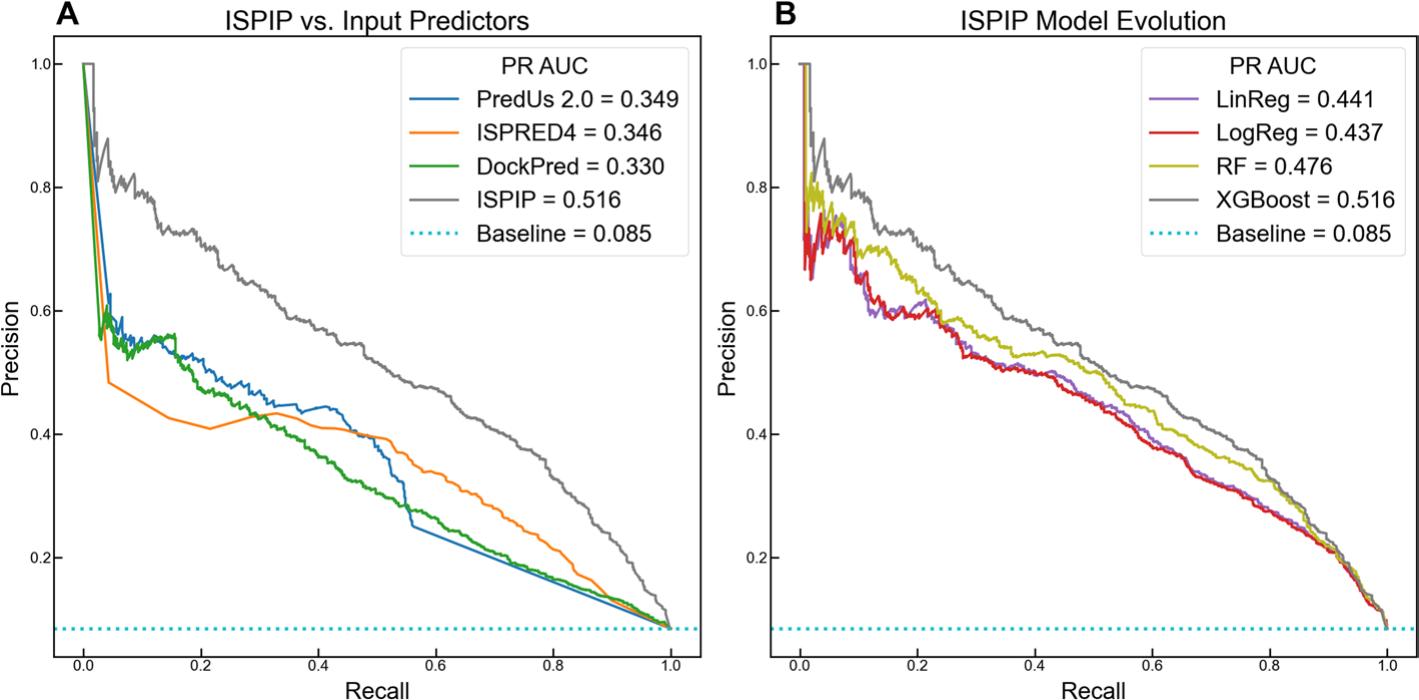
Enhanced prediction as ISPIP model evolves: (**A**) The PR curves of the 3 input methods indicate that PredUs 2.0 and ISPRED4 perform slightly better than DockPred. (**B**) All the ISPIP models significantly outperform the input predictors, and PR-AUC is boosted as the model evolves from simple linear regression to more complex ensemble decision tree algorithms

The linear and logistic regression models that were initially constructed yielded enhanced interface classification of test set query proteins according to both single-threshold (Table 1 and threshold-free metrics (Fig. 2). The threshold employed for single-threshold evaluation was determined by a dynamic cutoff (see Methods section). In predicting the interfacial residues for the test set proteins from Set A, the logistic regression model generated an average F-score of 0.469, which is significantly higher than the F-scores generated by the input methods that range from 0.380 to 0.405. The one sample Kolmogorov–Smirnov test [33] showed that the F-scores from the different input methods and the integrated methods were not normally distributed. The two sample Kolmogorov–Smirnov test showed that the F-score distributions obtained by the individual classifiers relative to the integrated method are statistically significantly different with > 95% confidence. All these two sample Kolmogorov–Smirnov tests resulting in a *p*-value < 0.05 show that the improvements in F-scores and MCC scores of ISPIP methods over the individual classifiers are statistically significant. MCC is generally considered to be more informative than F-scores because it captures model performance on both the positive and negative classes, whereas F-score is the harmonic mean between precision and recall, which are metrics that relate to the positive class. The linear and logistic ISPIP models yielded MCC values of 0.433, which is statistically significantly higher than the MCC values generated by the input methods, ranging from 0.324 to 0.355.

**Table 1:** ISPIP predictive enhancement by single-threshold metrics

Classifier	Average F-score	Average MCC
PredUs 2.0	0.400	0.351
ISPRED4	0.405	0.355
DockPred	0.380	0.324
Linear regression	0.470	0.433
Logistic regression	0.469	0.433

ISPIP’s enhanced predictive capacity is also illustrated in the PR (Fig. 2B) and ROC (Additional file 1: Figure S1) curves, and in their corresponding AUC metrics. The linear model had a PR-AUC of 0.441, which is significantly greater than the PR-AUC of DockPred, ISPRED4, and PredUs 2.0 (0.330, 0.346, and 0.349 respectively). Figure 2A also includes the XGBOOST results for an easy comparison with the individual classifiers. Similar improved prediction results were obtained for Set B proteins shorter than 450 residues (Additional file 1: Figure S2). A small percentage of a protein’s residues appear at the interface, which makes interface prediction an imbalanced classification problem. PR curves are shown here because they are more informative than ROC curves for imbalanced datasets [34]**.** Nevertheless, the Supplementary Information (Additional file 1: Figures S1 and S2) show the improvement of ROC-AUC from the input methods (0.726 to 0.823) to the ISPIP linear model (0.881). The statistical significance of ISPIP’s enhanced ROC-AUC was confirmed using the STAR approach [35] (Additional file 2: Table S1).

### Evolution of ISPIP model

After establishing the efficacy of combining orthogonal methods through simple regression models, we sought to further enhance ISPIP’s predictive capability by using more complex decision tree algorithms, Random Forest (RF) and Gradient Boosted Trees (XGBOOST). After optimizing the parameters through a fivefold Cross Validation process, the trained RF and XGBOOST models were implemented to predict the interface likelihood of query protein residues in the test sets from Set A and B. For Set A, the RF prediction achieved an MCC of 0.458 (Table 2) and PR-AUC of 0.476 (Fig. 2B), while the XGBOOST prediction achieved an MCC of 0.487 and PR-AUC of 0.516. A similar improvement was also observed in the average F-scores (Table 2) and AUC-ROC (Additional file 1: Figure S3). Since the F-scores are not normally distributed, we used the two sample Kolmogorov–Smirnov tests which resulted in a *p*-value < 0.05 to show that the improvements in F-scores and MCC scores are statistically significant. The pattern of ISPIP’s predictive capacity being initially enhanced from the regression models to RF, and further improved from RF to XGBOOST, is observed in both single-threshold and threshold-free metrics.

**Table 2:** Increased performance with ISPIP model evolution

ISPIP model	Average F-score	Average MCC
Random forest	0.490	0.458
XGBoost	0.516	0.487

### Integration of orthogonal predictions to form a consensus prediction

**Fig. 3:**
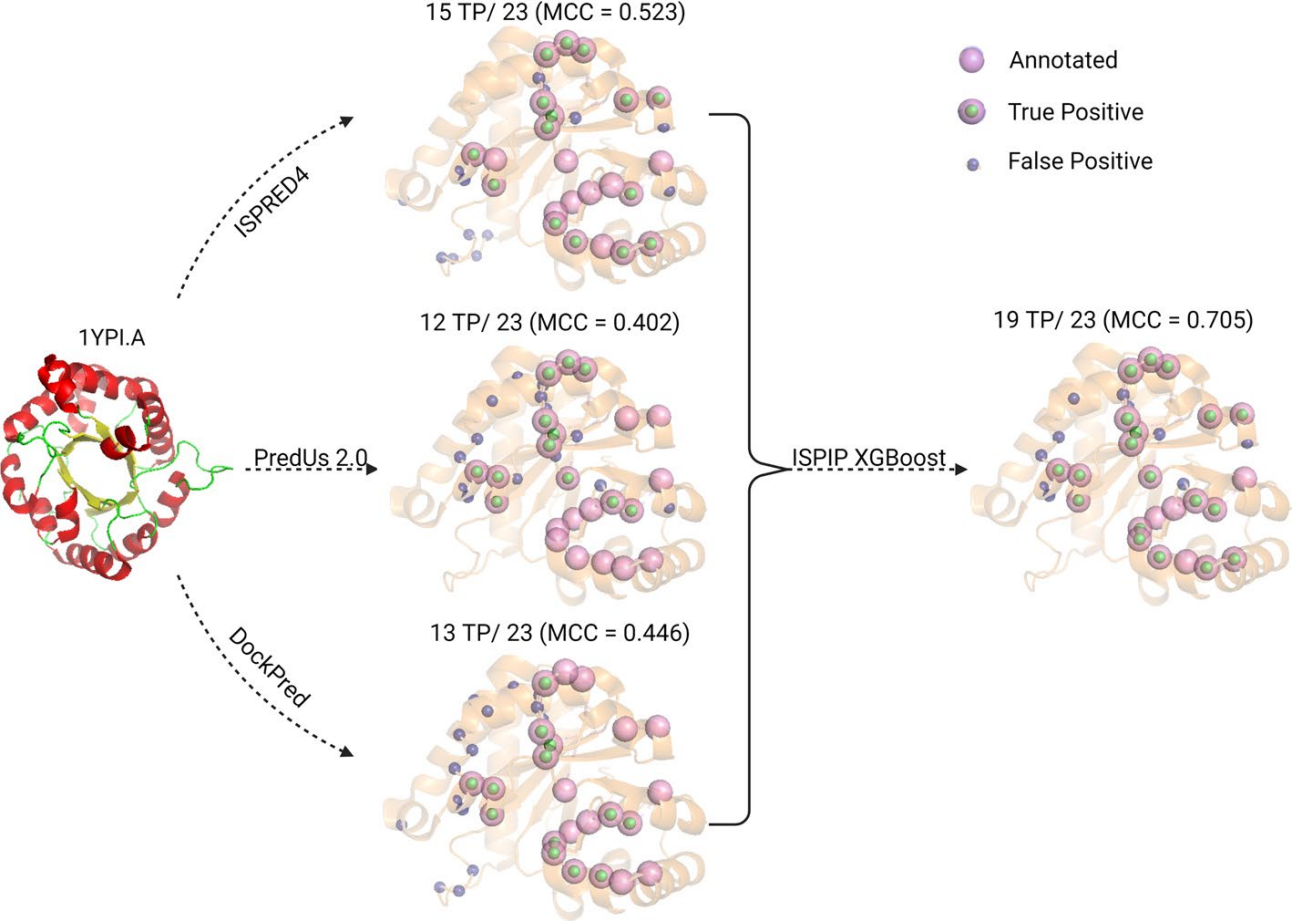
ISPIP consensus prediction of interface residues: On the left, the structure (1YPI.A) is shown. In the middle, the interface prediction of the 3 input classifiers is displayed. On the right, the ISPIP consensus prediction includes overlapping and unique TP residues of the input classifiers to yield an improved interface prediction of 19 TP out of the 23 annotated residues

Triosephosphate isomerase (1YPI.A) from *S. cerevisiae* is a query protein that illustrates how ISPIP integrates its three input methods to formulate an enhanced interface prediction. Out of the 23 annotated interfacial residues, PredUs 2.0 predicted 12 of them correctly (MCC = 0.402), DockPred predicted 13 of them correctly (MCC = 0.446), and ISPRED4 predicted 15 of them correctly (MCC = 0.523). The 3 predicted interfaces have 7 overlapping true-positive residues that are all present in the ISPIP interface prediction (Fig. 3). However, ISPIP is also able to retain additional, non-overlapping residues in its positive class to correctly predict a total of 19 of the 23 interfacial residues (MCC = 0.705).

### Set A vs B: including larger proteins in the dataset

Sulfite oxidase (1SOX.A) and *acetohydroxy acid isomeroreductase* (1YVE.I) were the two proteins larger than 450 residues that were added to Set B’s test set to form Set A’s test set. The optimized parameters for the 4 ISPIP regression models trained on Set B are included in the supplementary information (Additional file 2: Table S2). Only 1YVE.I was a significant outlier, as DockPred’s prediction generated a negative MCC score (– 0.061). The outlier slightly lowered the average MCC of DockPred from 0.336 in Set B to 0.324 in Set A, as well as ISPIP’s average MCC from 0.495 to 0.487 (Table 3). Since Set A includes proteins of all size*s* and it has MCC scores very close to Set B scores, Set A results have been reported as the default in this paper.

**Table 3:** Set A vs Set B model performance

	Set A: Average MCC	Set B: Average MCC
DockPred	0.324	0.336
XGBoost	0.487	0.495

## Discussion

### Linear vs. logistic regression

In general, linear regression models are suitable for cases where the target variable is continuous, and logistic models are appropriate for instances where the target variable is categorical. Since our target variable indicated whether a residue was experimentally determined to be at the interface or not (1 or 0), we expected the logistic model to have a greater predictive capacity than the linear model. Surprisingly, the logistic and linear models had almost identical F-scores (0.469 and 0.470, respectively) and PR-AUC values (0.437 and 0.441, respectively). This similarity in performance may indicate that three input variables are not enough to observe the difference in linear and logistic models, or perhaps it suggests that that the interfacial scores should be treated rather as a continuous variable in future studies.

### Performance comparison to VORFFIP and meta-PPISP

**Fig. 4:**
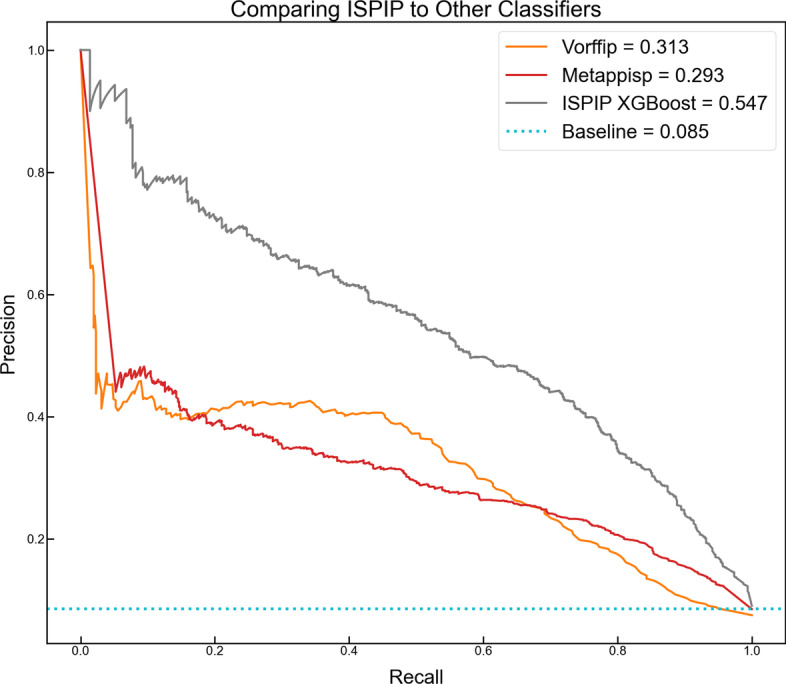
ISPIP outperforms other structure-based classifiers and meta-predictors: The PR curves highlight ISPIP’s improved performance of a complex structure-based classifier (VORFFIP) and previous meta-predictor (meta-PPISP)

We assessed the performance of ISPIP on the test set query proteins (Set A) relative to a top-performing structure-based method (VORFFIP) and the most recently available meta-method (meta-PPISP). Both methods base their predictions on the structure of the query protein and do not require structural information on a cognate partner. For this reason, as well as the availability and easy accessibility of their webservers, we chose to compare our results from ISPIP to these two methods. VORFFIP, developed by Segura et al. [36], uses a random forest method to integrate heterogeneous data including various residue level structural and energetic features, evolutionary sequence conservation, and crystallographic B-factor. Meta-PPISP is a metamethod that integrates the structure-based approaches cons-PPISP, Promate, and PINUP through linear regression. The most significant contrast in performance can be seen in the PR curves (Fig. 4), where ISPIP has as PR-AUC of 0.516 relative to VORFFIP’s score of 0.313 and meta-PPIPSP’s score of 0.293. Single-threshold metrics also confirm ISPIP’s (MCC = 0.487) superior predictive capacity relative to meta-PPISP (0.295) and VORFFIP (0.301).

### Driving factor of ISPIP’s enhanced performance

One primary question about ISPIP was whether its enhanced performance was due to the best performing input method (ISPRED4) or a result of the classifier integration process. To investigate this, consensus models were trained on only 2 of the 3 input predictors on the Set A proteins. The PR-AUC of DockPred-PredUs 2.0 model was 0.421 (Additional file 1: Figure S4), which was significantly greater than any of the 3 individual classifiers (PR-AUC = [0.330, 0.346]). This shows that the integration process itself, without the presence of ISPRED4, already generates an enhanced predictor. Replacing PredUs 2.0 with ISPRED4 does improve the DockPred-ISPRED model (PR-AUC = 0.448), so the identity of the input predictors does have some effect on the final model. However, the main driver of ISPIP’s enhanced prediction is the integration process, which can be seen when all 3 classifiers are combined to generate the complete ISPIP model with PR-AUC = 0.516.

### Robustness of ISPIP

**Fig. 5:**
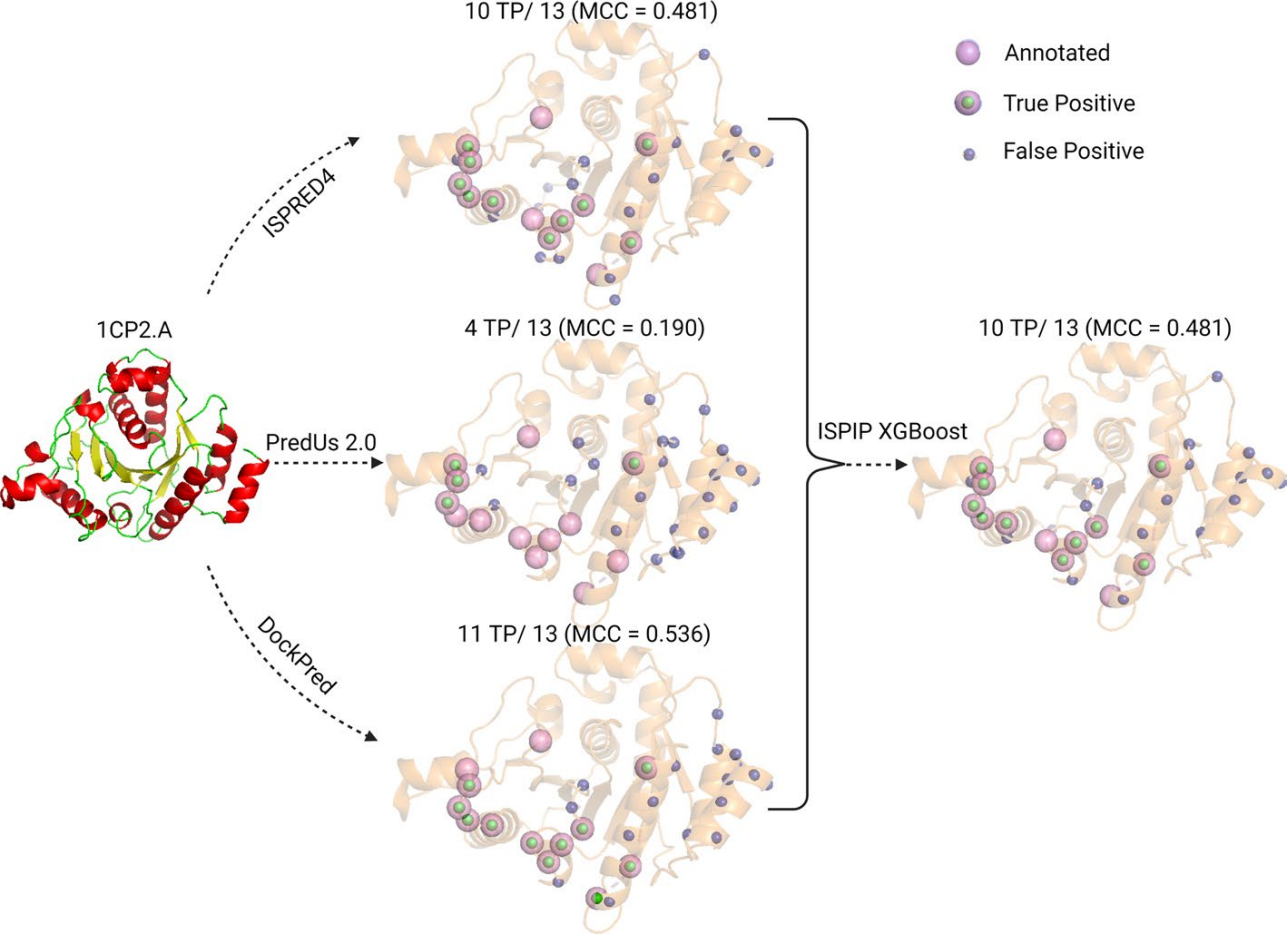
ISPIP is robust to poor performance of input classifier: On the left, the structure of 1CP2.A) is shown. In the middle, the interface prediction of the 3 input classifiers is displayed. PredUs 2.0 has an especially poor prediction relative to the other 2 input classifiers. On the right, the ISPIP has a robust consensus prediction with 10 TP out of the 13 annotated residues, despite the poor performance of the PredUs 2.0 input classifier

Nitrogenous iron protein (1CP2.A) from *C. pasteurianum* is a query protein that illustrates how ISPIP’s predictive model is robust to one of its input methods performing poorly (Fig. 5). Out of the 13 annotated interfacial residues, DockPred predicted 11 of them correctly (MCC = 0.536) and ISPRED4 predicted 10 of them correctly (MCC = 0.481); however, PredUs 2.0 was only able to predict 4 interfacial residues correctly (MCC = 0.190). It is very likely that the underperformance of PredUs 2.0 is due to a dearth of structural neighbors with an experimentally determined complex structure for 1CP2.A. ISPIP is able to integrate the input predictors in a robust manner to correctly predict 10 of the 13 interfacial residues, despite the poor performance of PredUs 2.0.

## Conclusions

There are currently several structure-based methods for predicting interfaces of proteins that can be categorized as either template-based or template-free. The performance of template-based methods is limited by the availability of template complex structures, and template-free approaches are limited by the number of biologically relevant features that can be included in the model. We show that these performance limitations can be overcome by using a method that integrates the orthogonal properties of a template-based, template-free, and docking-based approaches for consensus prediction of protein interfaces by using effective machine learning algorithms. A dataset of 156 proteins chosen from the docking benchmark and NOX benchmark with less than 30% sequence similarity, ranging in size from < 100 residues to about 600 residues and representing various CATH superfamilies were used for training and testing ISPIP. We demonstrate that more complex machine learning algorithms like random forest and gradient boosted trees perform better than the simpler linear or logistic regression models. All of these integrated models perform better than the best performing individual classifier. The integrated method is robust even when one of the individual classifiers performs poorly on a query protein. Since it is often not possible to predict which individual classifier will perform better on a given query protein, using an integrated approach for interface prediction should have a greater chance of success at predicting protein interfaces.

## Methods

### Development of ISPIP

In our previous work [32], we reported a docking-based interface classifier that is referred to here as DockPred. To enhance DockPred’s predictive capability, we have developed a meta-classification model that integrates DockPred with orthogonal template-free (ISPRED4) and template-based (PredUs2.0) classifiers. This was accomplished using the three methods as inputs to train various regression models on the training set described below, and then using the trained models with optimized parameters to classify interface residues for the query proteins in the test set (Fig. 1).

### The three input classifiers used in the development of the integrated method, ISPIP, are briefly described below

The first version of PredUs, developed in 2011, makes interface predictions for a query protein based on the known binding interfaces of the query’s structural neighbors. An improved version (PredUs 2.0) was developed in 2015 by adding sequence information to the template-based prediction. Using a Bayesian approach, PredUs 2.0 combines an amino acid interface propensity score with the template-based score of PredUs [37]. The original PredUs program uses the structural alignment program Ska [38] to identify a query protein’s structural neighbors and a structural alignment score is calculated [39]. Structural neighbors with a sequence similarity larger than 40% are identified using cd-hit [40] and retained. For every structural neighbor retained, PredUs calculates a contact frequency for each residue in the query protein by relating the structural neighbor’s binding partner to the query protein. This is then weighted by the closeness of the structural neighbor to the query protein. PredUs uses a support vector machine (SVM) algorithm to generate its template-based prediction score [1]. PredUs 2.0 includes information on the interface propensity values of the residues to calculate an interface probability score for each query residue.

ISPRED4 is one of the best performing template-free protein binding interface predictors currently available. It was developed by training an SVM model on a dataset (DBv5Sel) of 314 different monomer chains with complex structures that had been resolved by X-ray crystallography. Interface residues are defined as those that lost at least 1 Å^2^ of Accessible Surface Area (computed with the DSSP program [41]) when transitioning from a protein’s unbound to complex form. In the SVM model, each of the training proteins’ surface residues is represented by a 46-dimensional feature vector consisting of 10 different groups of descriptors. The feature vector included 34 sequence-based features that formed 5 groups of descriptors that included evolutionary information. The feature vector also included 12 structure-based features that comprised 5 groups of descriptors. ISPRED4 combines its SVM model with a Grammatical-Restrained Hidden Conditional Random Field (GRHCRF) to account for possible correlations between neighboring surface residues. For a given query protein, ISPRED4 calculates interface prediction scores by plugging the query residues’ feature vectors into its trained SVM/GRHCRF model [24].

DockPred demonstrated our previous hypothesis [42] that both substrate and non-substrate small organic molecules have a tendency to bind to similar, energetically favorable sites on a target protein (“sticky” sites) regardless of their biological relevance, also applies to the binding of proteins. The query protein is docked on 13 different non-cognate partner proteins that vary in size and represent different protein folds (immunoglobulin, and other small protein folds). The success of DockPred showed that non-cognate protein ligands preferentially bind to the cognate binding site of a target protein [32]. DockPred generates 2000 docked poses for each of the 13 binding partners using ZDOCK [43] or GRAMM [44]. The query protein residues are each assigned a probability to be at an interface by taking the average number of times a residue appears at the interface of 2000 docked poses for each of 13 different binding partners. A residue is considered to be at the interface of a docked pose if any atom of this residue is within 4.0 A of any atom of the binding partner and if the contact was considered legitimate according to the CSU program [45].

### Dataset

The dataset originally employed to test DockPred’s performance consisted of 233 unbound protein structures from the Docking Benchmark version 5 [46] and NOX [47] databases. Each protein had an unbound structure, as well as a corresponding complex structure available from the Protein Data Bank (PDB) [9]. The CATH superfamily classification exists for 91 of the proteins in this dataset. The major CATH superfamilies represented are Immunoglobulin like (33 proteins), Rossman fold (20 proteins). The other superfamilies like TIM Barrel, four helix, OB fold and Jelly roll are also represented. The true interface residues, or annotated residues, were determined by the CSU program [45] to find the legitimate contacts between query proteins and their complex partner. Residues of interacting proteins were classified as part of the interface if at least one complementary atom contact was detected by CSU within 4.0 Å of the partner protein, and if the contact was legitimate as defined by Sobolev, et al.

### Training and test sets

To eliminate redundancy of query proteins in the test and training sets, 156 proteins with less than 30% sequence similarity to all other proteins, as determined by Clustal Omega [48], were retained. The dataset contained proteins of sequence length ranging from 50 to 800 residues. Due to the challenges of docking larger proteins, DockPred’s predictive capacity significantly declined for proteins larger than 450 residues. Hence, we split the dataset into two subsets. One subset (set A) contained all 156 proteins, while the other subset (set B) consisted of the 141 proteins with fewer than 450 residues. 31 of the proteins from set B were randomly assigned to the test set, and the remaining 110 were split into 5 cross-validation (CV) subsets of 22 proteins each. The 15 proteins that contain more than 450 residues were randomly added to these test and training sets to form the set A training and test sets. The CATH superfamilies described above are well represented by both training and test sets.

To assess the extent of overlap between our test set and the training sets of the individual classifiers, the pairwise sequence identities between our test set and the training sets of the two individual classifiers, ISPRED4 and PredUs 2.0, were calculated. We observed that of a total of 90,486 pairwise alignments, 13 pairs (< 1%) had a sequence identity of > 35% with a member of the training sets of ISPRED4 or PredUs 2.0. We recalculated the average F-score and MCC score after removing these 13 proteins from our test set which had > 35% sequence identity with one of the proteins in the training sets of either ISPRED4 or PRedUs2.0. The average F-score and MCC score changed slightly, from 0.516 to 0.542 and from 0.487 to 0.503, respectively. All the sequence identity data are included in the Additional file 2: S3–S4 and Additional file 1: Figure S5 and S6.

## Machine learning algorithms

### Regression models

Using the three different classifiers, DOCKPRED, PredUS2.0 and ISPRED4, a normalized score between 0 and 1 was calculated for each residue for every protein in the training sets. This score represented the likelihood of a residue to be at the binding interface, as determined by each of the three classifiers. The three interface likelihood scores served as input variables $\left( {x_{1} ,x_{2} ,x_{3} } \right)$ for the linear and logistic regression models. The annotated score for each residue (0 = non-interfacial, 1 = interfacial) served as the target variable for the regression models. The logistic model parameters $\left( {b_{1} ,b_{2} ,b_{3} } \right)$ were fit by maximum likelihood estimation according to the function:

1 $$P\left( {i{|}x_{1} ,x_{2} ,x_{3} } \right) = \frac{1}{{1 + e^{{ - (b_{0} + \mathop \sum \nolimits_{j = 1}^{3} b_{j} x_{j} )}} }}$$

where ***P***(i|x_1_,x_2_, x_3_) is the probability that a residue, given the values of x_j_, will be in one of two discrete categories (interfacial or non-interfacial).

The linear model parameters $\left( {b_{1} ,b_{2} ,b_{3} } \right)$ were fit by ordinary least squares according to the function:

2 $$I\left( {x_{1} ,x_{2} ,x_{3} } \right) = b_{0} + \sum\nolimits_{{j = 1}}^{3} {b_{j} } x_{j}$$

where $I\left( {x_{1} ,x_{2} ,x_{3} } \right)$ is the continuous interfacial likelihood between 0 and 1. probability that a residue, given the values of x_j_, will be in one of two discrete categories (interfacial or non-interfacial).

The regression parameters $\left( {b_{1} ,b_{2} ,b_{3} } \right)$ were optimized using a fivefold (CV) scheme. Of the 5 sets in the training set each with 22 proteins (Set B) or 24 proteins (Set A), 4 sets were used for training and the fifth set served as the test set for the CV process. The model parameters were determined as the ones that generated the highest average F-score on the test CV subset (Table 4). These optimized parameters were then used to generate the interface probabilities for the proteins in the test set.

**Table 4:** Optimized regression parameters for Set A proteins

Regression model	PredUs 2.0 (*b*_1_)	ISPRED4 (*b*_2_)	DockPred (*b*_3_)
Linear	0.196	0.313	0.313
Logistic	1.28	2.821	1.424

### Ensemble decision tree algorithms: random forest and XGBOOST

The first ensemble decision-tree model that integrated the interface likelihoods from the three input classifiers was the random forest (RF) algorithm [49, 50]. At each node of the tree in the RF model, the classifiers and the cutoff values, for each classifier and each level of the tree, were chosen to optimize the results. The parameters representing the ensemble of trees in the forest, the maximum number of levels for each tree, and a tree pruning parameter, α, which chooses the subtree that minimizes the cost complexity measure, were all optimized to find the best fitting model with the three classifiers. The values for the optimized parameters are shown in Additional file 2: Table S2 and these were optimized to yield the best values for the average F-score, calculated as described below. For all the RF calculations, the optimal values of 100 trees, 10 levels and a pruning parameter of zero were used. Once again, a five-fold CV was used to obtain the optimized parameters. Once the trees are trained using the training set, the random forest model classifies the residues in the test set to one of the terminal nodes (leaves) in each tree in the forest. Based on the results from the training set, the probability of being an interface residue is calculated for each terminal node in each tree. A similar probability value was calculated for each terminal node for every tree in the random forest. Finally, the probability of being an interface residue was calculated as the average probability of all the terminal nodes in the forest into which the test residue is classified.

The other tree-based model employed for ISPIP involved gradient boosting. Like RF, gradient boosting constructs an ensemble of decision trees to generate an interface probability score. However, unlike RF, each successive tree produced by the gradient boosted algorithm “learns” from the previous trees in the forest by addition of a loss function and regularization parameter. Specifically, Histogram-based Gradient Boosting (XGBoost) was used to improve the speed and accuracy of the Decision Tree based regressor. A five-fold CV procedure was used to determine the optimal loss-function by maximizing the average F-score. The optimized parameters (Additional file 2: Table S2) were used to generate the interface probabilities for every residue for proteins in the test set as was done with the other models.

### Single-threshold evaluation of interface classifiers

The ISPIP method using regression, RF, and XGBOOST models generates interface likelihood scores (*p)*, between 0 and 1 for every residue of the proteins in the test set. To implement ISPIP as a classification model, each residue needs to be designated as interfacial (positive class) or non-interfacial (negative class). This can be based on a threshold value (*p*_*thr*_*)* such that residues with *p* > *p*_*thr*_ can be classified as interface residues. Another approach would be to choose a set number, N, of top-ranking residues (based on the *p* value) for every protein in the test set. This approach chooses a single threshold value, *N*, for each query protein. Zhang et al*.* [1] proposed a dynamic cutoff to determine N for each query protein according to the following equation:

3 $$N = 6.1\;R^{{0.3}}$$

where R referred to the number of the protein’s surface-exposed residues. Using this threshold value, the elements of the Confusion matrix, True Positive (TP), True Negative (TN), False Positive (FP) and False Negative (FN) can be determined. The binary classifier evaluation metrics used in this work are shown in Table 5. We used the nonparametric Kolmogorov–Smirnov (KS) single sample test to determine if the F-scores were normally distributed. For non-normal distributions, we used the two sample KS test to determine if the null hypothesis, that the distributions from the individual classifiers and the integrated method are the same, is valid.

**Table 5:** Binary classifier evaluation metrics

Precision = $$\frac{TP}{{TP + FP}}$$
Recall = True Positive Rate (TPR) = $\frac{TP}{{TP + FN}}$
False Positive Rate (TPR) = $\frac{FP}{{FP + TN}}$
F-Score = $$\frac{2*Precision*Recall}{{Precision + Recall}}$$
MCC = $$\frac{TP*TN - FP*FN}{{\sqrt {\left( {TP + FP} \right)\left( {TP + FN} \right)\left( {TN + FP} \right)\left( {TN + FN} \right)} }}$$

### Threshold free evaluation metrics—AUC under ROC and PR curves

Receiver Operator Characteristic (ROC) curves were generated using python’s scikit package [49] by plotting the true positive rate (TPR) vs the false positive rate (FPR) for different threshold values of *p*, ranging from 0 to 1. Precision-recall (PR) curves were generated with the same package by plotting the precision vs recall for different threshold values of *p*, ranging from 0 to 1. The area under the curve (AUC-ROC and AUC-PR) was calculated using the trapezoidal method.

We assessed the statistical significance of the differences in AUC-ROC for the different methods using the STAR software [35] uses the chi-squared distribution to test the null hypothesis that there is no difference between the AUC-ROC curves originating from the different methods. The difference between any two methods is assessed at a significance level of 0.05.

## Supplementary Information

**Additional file 1**. Supplementary Figures.

**Additional file 2**. Supplementary Tables.

## Data Availability

ISPIP is implemented in python and the code and sample data can be freely accessed from GitHub repository: https://github.com/eved1018/ISPIP.
