# Supplementary material for: Integrated structure-based protein interface prediction
Source: BMC Bioinformatics. 2022 Jul 25;23:301. doi: 10.1186/s12859-022-04852-2 (PMC9316365; doi:10.1186/s12859-022-04852-2)
Supplement: Supplementary file 1 — Additional file 1. Supplementary Figures. [file 12859_2022_4852_MOESM1_ESM.docx]

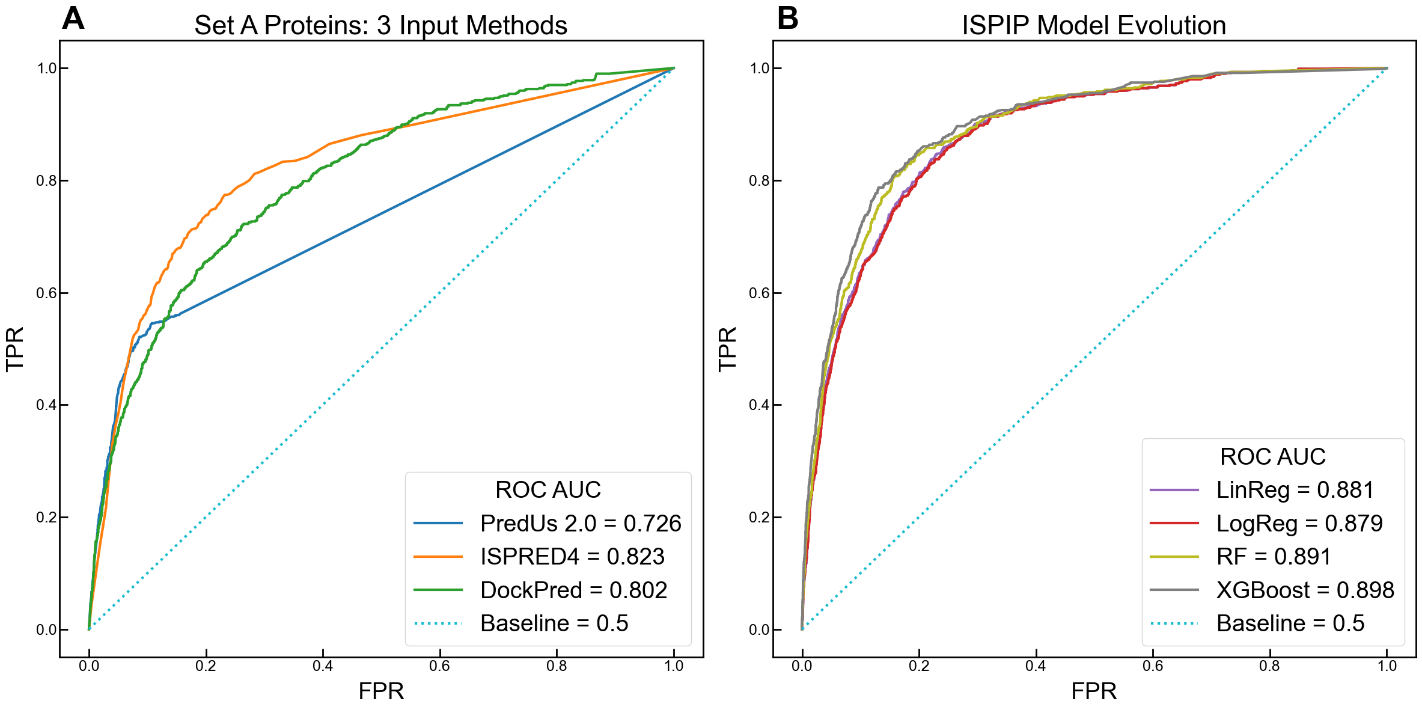


**Supplementary Figure 1. ROC illustrates enhanced ISPIP’s enhanced prediction on Set A proteins: (A)** The ROC curves of the 3 input methods indicate that DockPred and ISPRED4 perform better than PredUs 2.0. **(B)** All the ISPIP models significantly outperform the input predictors, and ROC-AUC is boosted as the model evolves from simple linear regression to more complex ensemble decision tree algorithms.


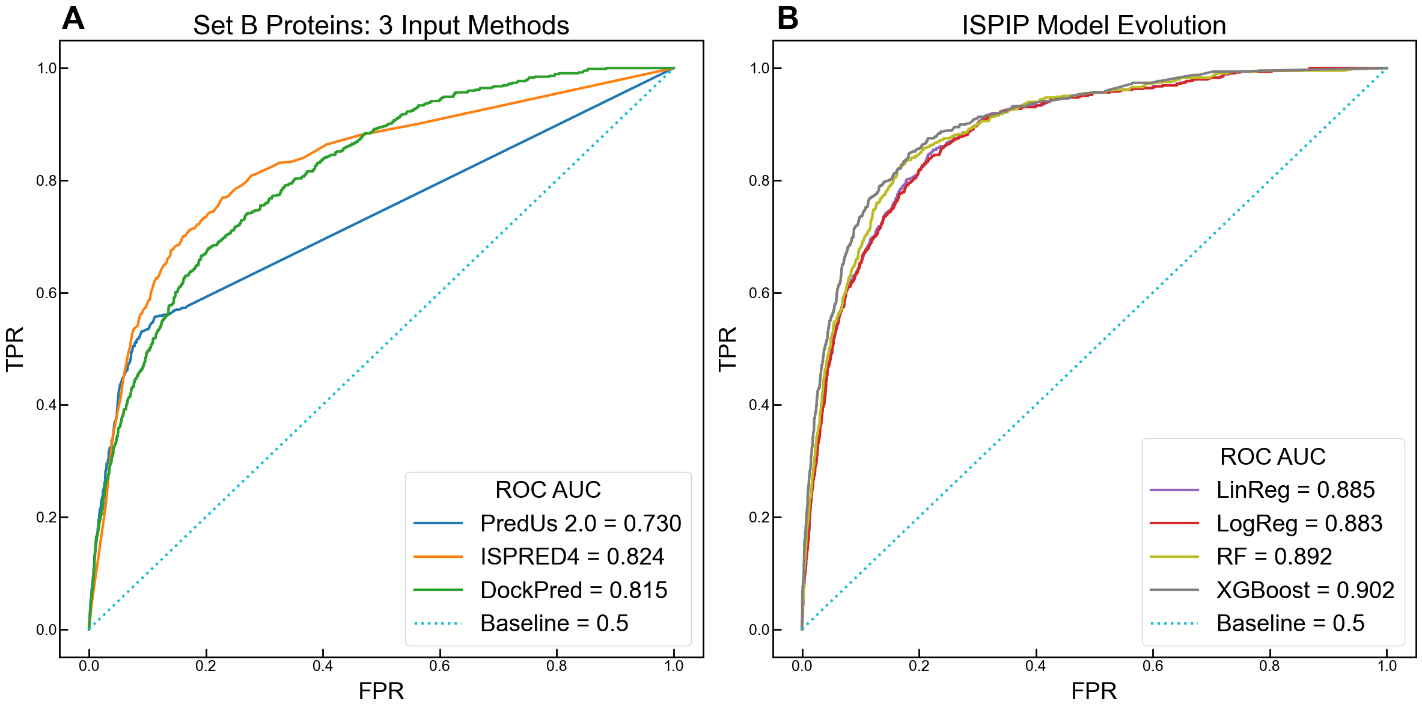


**Supplementary Figure 2. ROC illustrates enhanced ISPIP’s enhanced prediction on Set B proteins: (A, B)** Similar, but slightly improved, model improvement was observed for Set B proteins, from which proteins larger than 450 residues were removed.


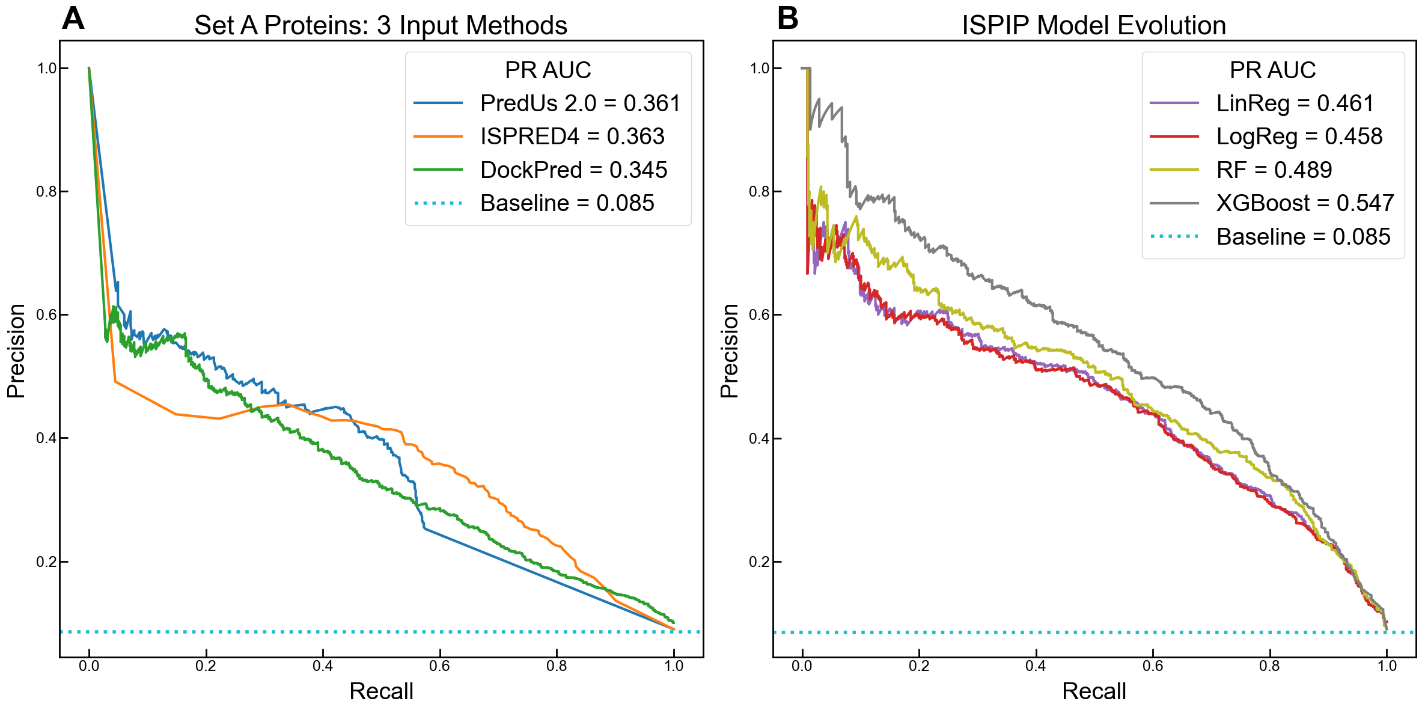


**Supplementary Figure 3. PR illustrates enhanced ISPIP’s enhanced prediction on Set B proteins: (A, B)** The model improvement observed for Set A proteins through the PR-AUC (Figure 1) can also be seen for Set B proteins.


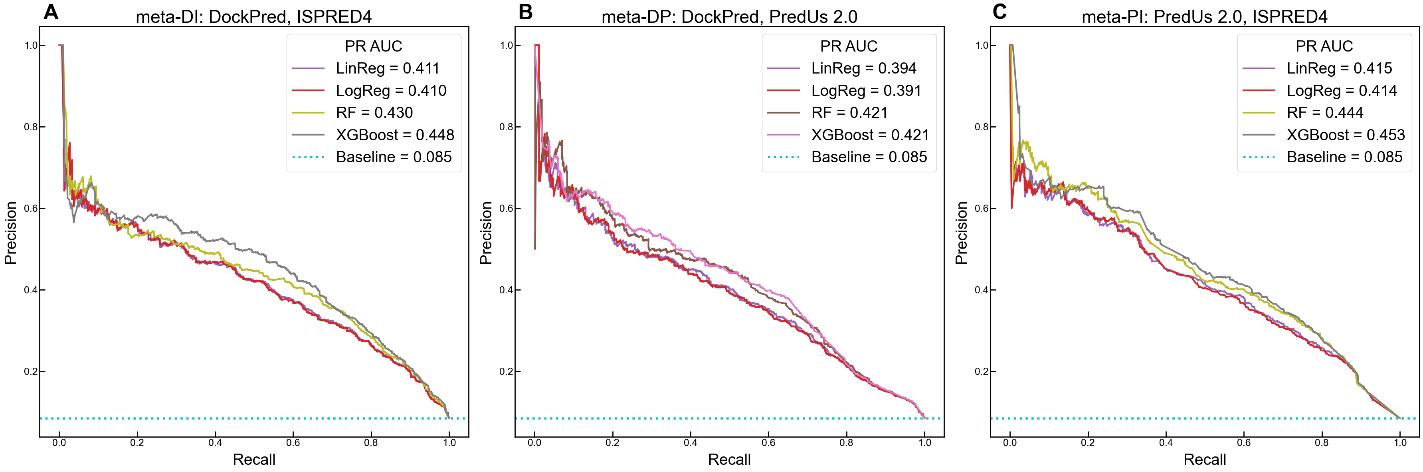


**Supplementary Figure 4. ISPIP models trained only on 2 of the 3 input classifiers: (A)** Similar to the complete ISPIP model, the one trained on only DockPred and ISPRED4 showed enhanced performance with increasing model complexity. **(B)** Method combination yields enhanced prediction, even without being trained on ISPRED4. **(C)** The model trained on ISPRED4 and PredUs 2.0 performs the best out of the models trained on 2 of the 3 input classifiers.

A B

- B

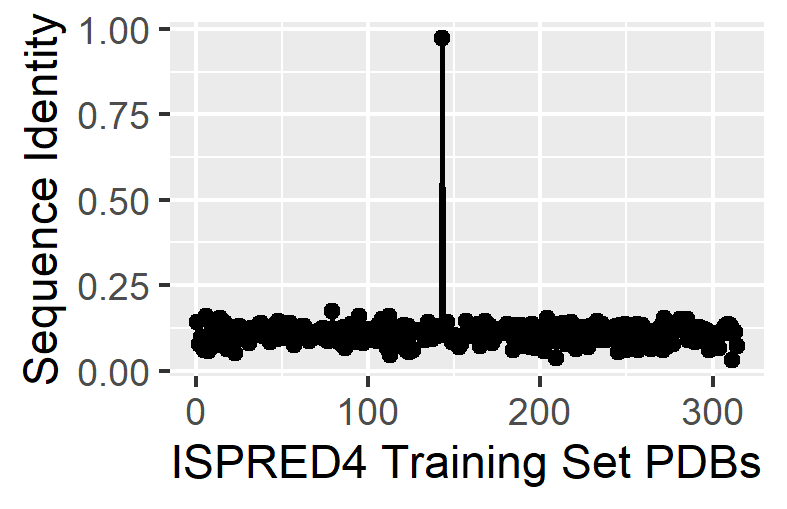

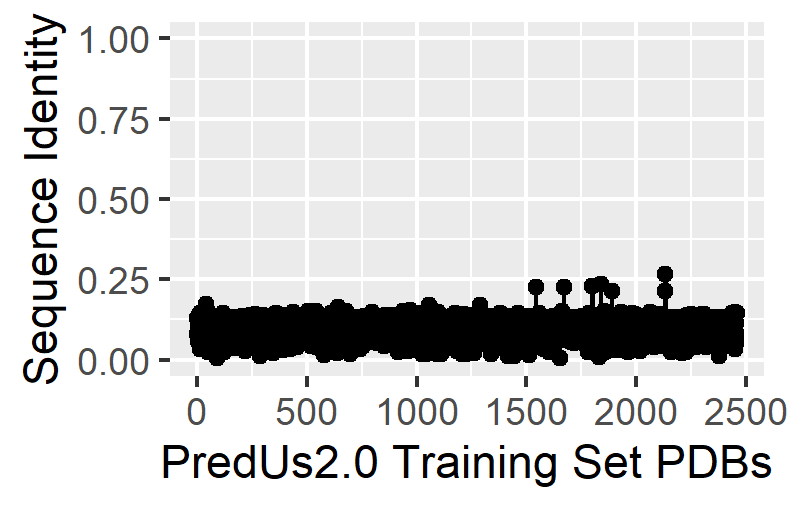


Supplementary Figure 5: Sequence identify distribution of a representative test set protein, 1KXP_D, with the training sets of ISPRED4 (A) and PredUs 2.0 (B).

A B


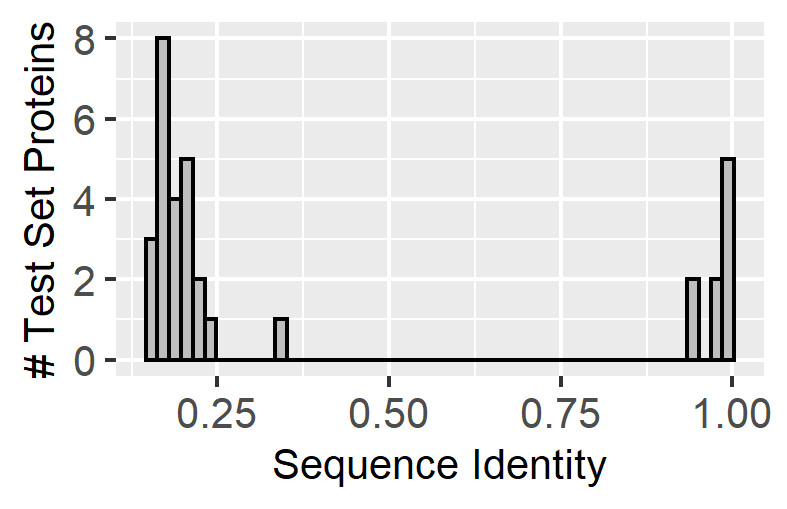

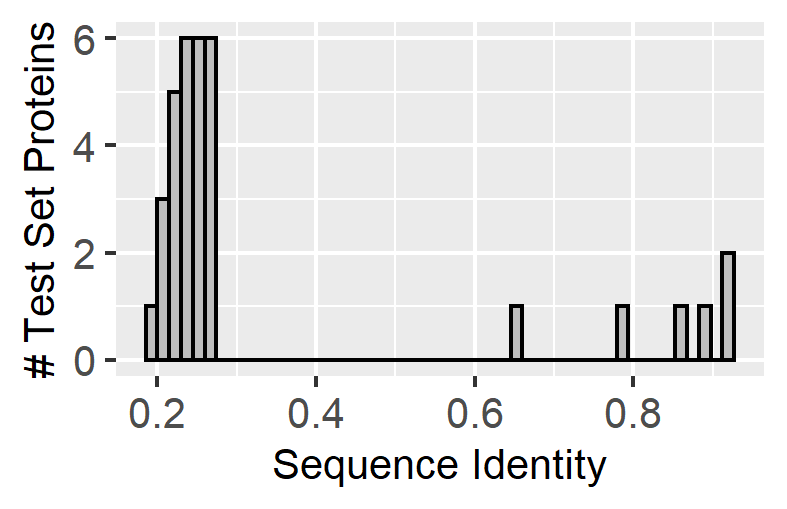


Supplementary Figure 6: Histogram of maximum sequence identify distribution of the ISPIP test set proteins with the training sets of ISPRED4 (A) and PredUs 2.0 (B).
