# Supplementary material for: Integrated structure-based protein interface prediction
Source: BMC Bioinformatics. 2022 Jul 25;23:301. doi: 10.1186/s12859-022-04852-2 (PMC9316365; doi:10.1186/s12859-022-04852-2)
Supplement: Supplementary file 2 — Additional file 2. Supplementary Tables. [file 12859_2022_4852_MOESM2_ESM.docx]

Table S1. Statistical significance of AUC-ROC differential between classifiers

|  | PredUs 2.0 | ISPRED4 | DockPred | LogReg | LinReg | RF | XGBoost |
| --- | --- | --- | --- | --- | --- | --- | --- |
| PredUs 2.0 | - | -0.096 | -0.075 | -0.152 | -0.154 | -0.165 | -0.171 |
| ISPRED4 | 1.78e-10 | - | 0.021 | -0.056 | -0.058 | -0.069 | -0.075 |
| DockPred | 3.37e-15 | \| 0.065 \|  \| \| --- \| --- \| | - | -0.077 | -0.079 | -0.09 | -0.096 |
| LogReg | 1.17e-54 | 5.46e-32 | 2.02e-19 | - | -0.002 | -0.013 | -0.019 |
| LinReg | 2.03e-58 | 9.55e-32 | 1.89e-20 | 2.03e-9 | - | -0.011 | -0.017 |
| RF | 3.87e-72 | 1.67e-26 | 5.32e-30 | 1.25e-8 | \| 2.62e-7 \| \| --- \| | - | -0.006 |
| XGBoost | 1.21e-72 | 1.10e-29 | 2.12e-30 | 1.35e-12 | 4.19e-11 | -3.48 e-11 | - |

Upper triangle displays difference between AUC-ROC of two given classifiers. Lower triangle displays p-value for statistical significance of AUC-ROC difference.

Table S2. Optimized regression parameters for Set B proteins

| Regression Model | PredUs 2.0 $(b_{1})$ | ISPRED4 $(b_{2})$ | DockPred $(b_{3})$ |
| --- | --- | --- | --- |
| Linear | 0.178 | 0.266 | 0.192 |
| Logistic | 1.27 | 2.519 | 1.764 |

Table S3. Maximum Sequence Identity of ISPIP Test Set and ISPRED4 Training Set

| ISPIP TEST PDB | ISPRED4-Training PDB with maximum sequence identity | Sequence Identity |
| --- | --- | --- |
| 1PDK_A | 1E6O_L | 0.1788 |
| 1CNZ_A | 2QA9_E | 0.163 |
| 1KXP_D | 1KW2_B | 0.9719 |
| 1COZ_A | 1JVM_A | 0.1794 |
| 1CLI_A | 3QI0_A | 0.1708 |
| 3BX7_A | 3BX8_A | 0.9765 |
| 1DE4_E | 1EGL_A | 0.213 |
| 2PCB_A | 1CCP_A | 0.9983 |
| 1B8A_A | 1HCL_A | 0.1585 |
| 1EG9_A | 1CCP_A | 0.1649 |
| 1DOW_A | 1SYQ_A | 0.2004 |
| 1STF_E | 3BPF_A | 0.349 |
| 2B4J_C | 1CEW_I | 0.2 |
| 1E4K_C | 1FNL_A | 0.9913 |
| 1DOR_A | 2C8B_X | 0.1836 |
| 1SOX_A | 2YVF_A | 0.1759 |
| 1PVH_B | 1EMR_A | 0.9512 |
| 1YVE_I | 1A12_A | 0.1597 |
| 2NAC_A | 1GJR_A | 0.1614 |
| 1QFH_A | 1SUP_A | 0.1889 |
| 1QOR_A | 3VL8_A | 0.1828 |
| 1HSS_A | 2UUX_A | 0.2169 |
| 3VLB_B | 3VL8_A | 0.9932 |
| 1JTD_A | 1ZG4_A | 0.9943 |
| 1TMQ_B | 1QFK_L | 0.216 |
| 2SIC_I | 3SSI_A | 0.9953 |
| 2UTG_A | 2J5Y_A | 0.2443 |
| 2CFH_C | 2BJN_A | 0.9416 |
| 1YPI_A | 3F74_A | 0.1827 |
| 1I8L_A | 3RVT_D | 0.2043 |
| 1CP2_A | 1HUR_A | 0.1782 |
| 1CMB_A | 1UNK_D | 0.2094 |
| 1TCO_A | 1IAM_A | 0.1676 |

Table S4. Maximum Sequence Identity of ISPIP Test Set and PredUs2.0 Training Set

| ISPIP TEST PDB | PredUs Training PDB with maximum sequence identity | Sequence Identity |
| --- | --- | --- |
| 1PDK_A | 2W07_A | 0.8903 |
| 1CNZ_A | 1JFI_B | 0.2569 |
| 1KXP_D | 1JFI_B | 0.2651 |
| 1COZ_A | 2B9S_A | 0.2319 |
| 1CLI_A | 1JFI_B | 0.2521 |
| 3BX7_A | 4GH7_A | 0.6541 |
| 1DE4_E | 4H25_E | 0.2067 |
| 2PCB_A | 1JFI_B | 0.2563 |
| 1B8A_A | 1JFI_B | 0.2444 |
| 1EG9_A | 2BMO_A | 0.7804 |
| 1DOW_A | 1JFI_B | 0.2216 |
| 1STF_E | 3IMA_A | 0.9123 |
| 2B4J_C | 2B9S_A | 0.2086 |
| 1E4K_C | 2B9S_A | 0.2292 |
| 1DOR_A | 1JFI_B | 0.2511 |
| 1SOX_A | 1JFI_B | 0.2607 |
| 1PVH_B | 2B9S_A | 0.24 |
| 1YVE_I | 1JFI_B | 0.2591 |
| 2NAC_A | 1JFI_B | 0.2631 |
| 1QFH_A | 2FO1_A | 0.2651 |
| 1QOR_A | 1JFI_B | 0.2388 |
| 1HSS_A | 1JFI_B | 0.2598 |
| 3VLB_B | 1JFI_B | 0.2438 |
| 1JTD_A | 3C7V_A | 0.9255 |
| 1TMQ_B | 1NVP_B | 0.2348 |
| 2SIC_I | 1N6J_A | 0.2593 |
| 2UTG_A | 2B9S_A | 0.1987 |
| 2CFH_C | 1JFI_B | 0.2123 |
| 1YPI_A | 1JFI_B | 0.2256 |
| 1I8L_A | 1JFI_B | 0.2164 |
| 1CP2_A | 1JFI_B | 0.267 |
| 1CMB_A | 2B9S_A | 0.227 |
| 1TCO_A | 4F0Z_A | 0.8543 |

Table S5. F-Score and MCC without the PDB with sequence identify > 35% with Training sets of ISPRED4 and PredUS2.0

|  | Test Set with 33 proteins | Test Set with 20 proteins |
| --- | --- | --- |
| Average F-score | 0.516 | 0.542 |
| Average MCC score | 0.487 | 0.503 |
